# Differences in Tumor‐Infiltrating Lymphocyte Counts in the Peritumoral Area in Patients Undergoing Hepatic Resection After Lenvatinib and Atezolizumab Plus Bevacizumab Therapy for Hepatocellular Carcinoma

**DOI:** 10.1002/cam4.70445

**Published:** 2025-04-18

**Authors:** Katsuya Toshida, Shinji Itoh, Yasushi Tanaka, Takeo Toshima, Shohei Yoshiya, Takuma Izumi, Norifumi Iseda, Yuriko Tsutsui, Yuki Nakayama, Takuma Ishikawa, Mizuki Ninomiya, Takeshi Iwasaki, Yoshinao Oda, Tomoharu Yoshizumi

**Affiliations:** ^1^ Department of Surgery and Science, Graduate School of Medical Sciences Kyushu University Fukuoka Japan; ^2^ Department of Anatomic Pathology, Graduate School of Medical Sciences Kyushu University Fukuoka Japan

**Keywords:** atezolizumab plus bevacizumab, conversion surgery, hepatocellular carcinoma, lenvatinib

## Abstract

**Aim:**

With advances in systemic therapy, the number of patients with hepatocellular carcinoma (HCC) who can undergo hepatic resection has increased in recent years, but there are no reports evaluating the immune status in the peritumoral area.

**Methods:**

We enrolled 14 patients who underwent hepatic resection after lenvatinib (LEN, *n* = 7) or atezolizumab plus bevacizumab (ATZ/BEV, *n* = 5) therapy. Tumor‐infiltrating lymphocytes (TILs), including CD3+ and CD8+ TILs, in the peritumoral area were evaluated by hematoxylin and eosin staining and immunohistochemistry.

**Results:**

The median TIL counts after LEN and ATZ/BEV therapy were 32 and 92 cells/0.237 mm^2^, respectively (*p* = 0.0044). The median CD3+ TIL counts after LEN and ATZ/BEV therapy were 26 and 71 cells/0.237 mm^2^, respectively (*p* = 0.0057). The median CD8+ TIL counts after LEN and ATZ/BEV therapy were 14 and 42 cells/0.237 mm^2^, respectively (*p* = 0.0044).

**Conclusion:**

TIL counts, including those of CD3+ and CD8+ TILs, in the peritumoral area were significantly higher after ATZ/BEV than after LEN therapy.

**Abbreviations**
ATZ/BEV: atezolizumab plus bevacizumab
HCC: hepatocellular carcinoma
LCR: lymphocyte‐C‐reactive protein ratio
LEN: lenvatinib
LMR: lymphocyte monocyte ratio
MRI: magnetic resonance imaging
NLR: neutrophil‐lymphocyte ratio
NMR: neutrophil‐monocyte
RECIST: Response Evaluation Criteria in Solid Tumors
TILs: tumor‐infiltrating lymphocytes
u‐HCC: unresectable hepatocellular carcinoma

## 1 Introduction

Hepatocellular carcinoma (HCC) is the fourth leading cause of cancer‐related death globally [1]. Much has been learned about the tumor microenvironment (TME) and molecular classification in HCC by researchers [2, 3]. From a therapeutic perspective, hepatic resection has been established as a safe, effective, and curative treatment. Meanwhile, systemic therapy has been developed at an ever‐increasing pace, and lenvatinib (LEN) and atezolizumab plus bevacizumab (ATZ/BEV) are widely used to treat unresectable HCC (u‐HCC) [4, 5]. However, the long‐term survival of patients with HCC remains suboptimal. In this situation, some patients experience tumor shrinkage or the disappearance of metastatic lesions after systemic therapy, thus meeting the criteria for resectability [6, 7, 8]. This treatment strategy, which aims to convert from u‐HCC to resectable HCC, is known as conversion therapy.

In hepatic resection, the remnant liver volume is extremely important because a small remnant liver volume might lead to liver failure [9]. For conversion surgery after systemic therapy in particular, the tumors are often large, and there is a need to proceed with liver resection at approximately the boundary between the tumor and normal liver to preserve as much residual liver as possible [10, 11]. Therefore, it is important for surgeons to understand the nature of the peritumoral area, including the inflammatory state. To the best of our knowledge, no reports have evaluated the immune status in the peritumoral area in patients who underwent hepatic resection after systemic therapy. In this study, we investigated the immune status in the peritumoral area of resected tumors in patients who underwent hepatic resection after LEN or ATZ/BEV therapy.

## 2 Materials and Methods

### 2.1 Patients

In this study, among 116 patients treated with lenvatinib (*n* = 75) or ATZ/BEV (*n* = 51) for u‐HCC from April 2018 to January 2023 at Kyushu University Hospital, 14 patients who had a partial response or complete response according to the modified Response Evaluation Criteria in Solid Tumors (RECIST) as the best response to LEN or ATZ/BEV and underwent hepatic resection were included. One patient with vv3 was excluded because of the presence of combined inferior vena cava resection. Eight patients who underwent hepatic resection after LEN therapy and five patients who underwent hepatic resection after ATZ/BEV therapy were included in the final evaluation. The detailed surgical procedure for hepatic resection has been previously described [12]. The time period of discontinuation of LEN therapy until surgery was one week and that of ATZ/BEV therapy was 6 weeks.

### 2.2 Regimens of Systemic Therapy

The dosage and administration of LEN were previously described. Patients who weighted < 60 kg received 8 mg/day LEN, and those weighing ≥ 60 kg received 12 mg/day LEN [13]. The dosage and administration of ATZ/BEV were previously described. Intravenous ATZ/BEV treatment consisted of 1200 mg of ATZ plus 15 mg/kg body weight BEV every 3 weeks [14]. Starting with a reduced dose was permitted depending on the patient's condition. Follow‐up visits for all patients included blood chemistry and tumor marker measurements. Attending clinicians and pharmacists checked for the presence and grade of adverse events for patients in each of their regular visits.

### 2.3 Clinical Data

Patient characteristics (hepatitis B surface‐antigen‐positive, hepatitis C virus antibody‐positive, preoperative treatment, administration period of systemic therapy, lymphocyte‐C‐reactive protein ratio (LCR), lymphocyte monocyte ratio (LMR), neutrophil‐lymphocyte ratio (NLR), neutrophil‐monocyte ratio (NMR), best response (RECIST/modified RECIST), surgical procedure, blood loss, surgical margin, pringle time, time of surgery, and gross classification) were recorded.

### 2.4 Gd‐EOB‐DTPA‐Enhanced Magnetic Resonance Imaging (MRI)

All magnetic resonance imaging (MRI) procedures were carried out using a whole‐body 3.0‐T scanner (Intera Achieva Nova Dual; Philips Medical Systems) with sensitivity‐encoding acceleration techniques using a 32‐channel phased‐array coil. For Gd‐EOB‐DTPA‐enhanced MRI, the recommended full dose of gadoxetic acid (EOB Primovist; Bayer Health‐ Care) according to bodyweight (0.1 mL/kg) was intravenously injected. A multiphase dynamic study including arterial, portal, and late phases was undertaken. The evaluation method of MRI was previously described [15].

### 2.5 Hematoxylin and Eosin Staining and Immunohistochemistry (IHC)

We stained sections with hematoxylin and eosin. Immunohistochemical staining was conducted as previously described [16, 17]. Formalin‐fixed, paraffin‐embedded tissue sections from patients with HCC were immunostained for mouse monoclonal anti‐CD3 (F7.2.38, Dako, Carpinteria, CA, USA), mouse monoclonal anti‐CD8 antibody (Clone C8/144B; Agilent Technologies, Santa Clara, CA, USA), mouse monoclonal anti‐β‐catenin antibody (Clone E‐5; Santa Cruz, CA, USA), mouse monoclonal anti‐glutamine synthetase antibody (66323‐2‐Ig; Proteintech, Rosemont, IL, USA), and rabbit polyclonal anti‐OATP1B3 antibody (HPA004943; Sigma‐Aldrich, MO, USA). The capture of microscopic images and quantitative analyses were undertaken on the NanoZoomer platform (Hamamatsu Photonics).

### 2.6 Evaluation Method of Tumor‐Infiltrating Lymphocytes (TILs)/CD3+ TILs/CD8+ TILs

**FIGURE 1:**
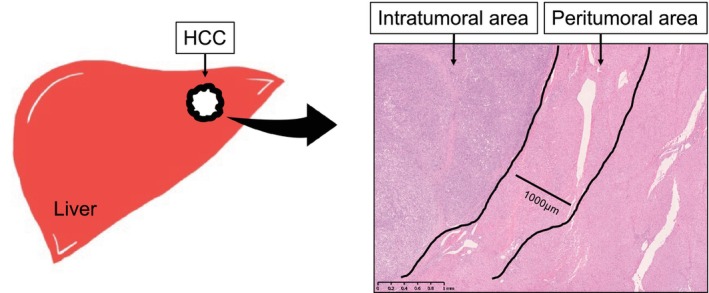
Definition of TILs in the peritumoral area of HCC. Peritumoral sites in the area within 1000 μm outside the outermost part of the edge of the tumor were analyzed in this study. HCC, hepatocellular carcinoma; TILs, tumor‐infiltrating lymphocytes.

The number of TILs was assessed using standardized methods for TIL analysis in solid tumors, and we assessed peritumoral sites, which were determined to be within 1000 μm outside the outermost part of the edge of the tumor, as presented in Figure 1 [18, 19]. If the tumor had a capsule, the outer edge of the capsule was defined as edge of the tumor. All sections obtained from each patient were reviewed using light microscopy (×400 magnification, 40× objective lens, and 10× ocular lens; 0.237 mm^2^ per field). The average numbers of TILs, CD3+ TILs, and CD8+ TILs were calculated for the five areas with the highest density of staining in the peritumoral area. Evaluations were performed independently by four independent observers (K.T., S.I., Y.T. and T.I) who were blinded to the clinical background of the subjected patients. The average of the counts by the three observers was used as the final count.

### 2.7 Statistical Analysis

Statistical analyses were performed using SAS software (JMP Pro 16; SAS Institute Inc.). The Shapiro–Wilk test was used to assess whether continuous variables were normally distributed. Continuous variables were presented as the median and compared using the Mann–Whitney U‐test. Categorical variables were reported as percentages and compared using the chi‐squared test or Fisher's exact test. Covariates that were significant at *p* < 0.05 were included in the multivariate Cox proportional hazards model.

## 3 Results

### 3.1 Perioperative Characteristics of the Patients

The perioperative characteristics of patients who underwent hepatic resection after LEN or ATZ/BEV therapy are summarized in Table 1. The median (range) duration of administration period was 14 (8–49) weeks for LEN therapy and 12 (7–30) weeks for ATZ/BEV therapy. The number of patients whose resection was greater than segmentectomy was five (71.4%) for LEN and four (80.0%) for ATZBEV. Information on preoperative biomarkers is described in Table S1. There were no obvious differences in blood loss, the Pringle time, and the duration of surgery between the two regimens.

**TABLE 1:** Perioperative characteristics of patients who underwent hepatic resection.

Case	Treatment	Etiology	Administration period	NLR before/after systemic therapy	Best response: RECIST modified RECIST	Surgical Procedure	Blood loss (ml)	Surgical margin (mm)	Pringle time (min)	Duration of surgery (min)
1	LEN	HBV‐Ag (+)	23 weeks	1.72/2.41	PR PR	Living donor liver transplantation	450	NA	—	471
2	LEN + TACE	HBV‐Ag (+)	28 weeks	3.68/1.04	PR PR	S8 segmentectomy of the liver S2 partial hepatectomy, MCT	140	10	71	276
3	LEN	HBV‐Ag (+)	49 weeks	1.30/2.25	PR PR	Laparoscopic S5 partial hepatectomy	60	3	23	110
4	LEN	nonBnonC	11 weeks	3.65/2.97	PR PR	Central bisectionectomy of the liver	835	5	135	510
5	LEN	nonBnonC	8 weeks	2.38/3.20	SD PR	Left hemihepatectomy Tumor thrombectomy	231	10	57	307
6	LEN	nonBnonC	9 weeks	0.84/1.64	PR PR	S7 + 8 segmentectomy S4 partial hepatectomy	1144	2	107	327
7	LEN + TACE	HCV	14 weeks	3.53/3.26	SD PR	Hepatic caudate lobectomy, partial hepatectomy S6/7, and MCT	1990	0	90	658
8	ATZ/BEV	HBV‐Ag (+)	30 courses	4.30/2.33	PR PR	Extended S7 segmentectomy of the liver	590	2	56	290
9	ATZ/BEV	nonBnonC	7 courses	3.57/3.69	PR CR	Central bisectionectomy of the liver	2307	0	180	486
10	ATZ/BEV	nonBnonC	12 courses	6.52/4.92	PR PR	Laparoscopic S5 partial hepatectomy and S6 segmentectomy of the liver	225	1	147	332
11	ATZ/BEV	HBV‐Ag (+)	10 courses	2.84/3.04	PR PR	Anterior sectionectomy combined with S6 partial resection of the liver	168	7	78	308
12	ATZ/BEV	HCV‐Ab (+)	15 courses	1.37/0.75	PR CR	Laparoscopic S6/7 partial hepatectomy	150	3	88	293

Abbreviations: ATZ/BEV, atezolizumab plus bevacizumab; CR, complete response; HBV‐Ag, hepatitis B surface‐antigen; HCV‐Ab, hepatitis C virus antibody; LEN, lenvatinib; MCT, microwave coagulation therapy; NA, not applicable; NLR, neutrophil‐lymphocyte ratio, RECIST, Response Evaluation Criteria in Solid Tumors; PR, partial response; TACE, transcatheter arterial chemoembolization.

### 3.2 Differences in TIL/CD3+ TIL/CD8+ TIL Counts in the Peritumoral Area

**FIGURE 2:**
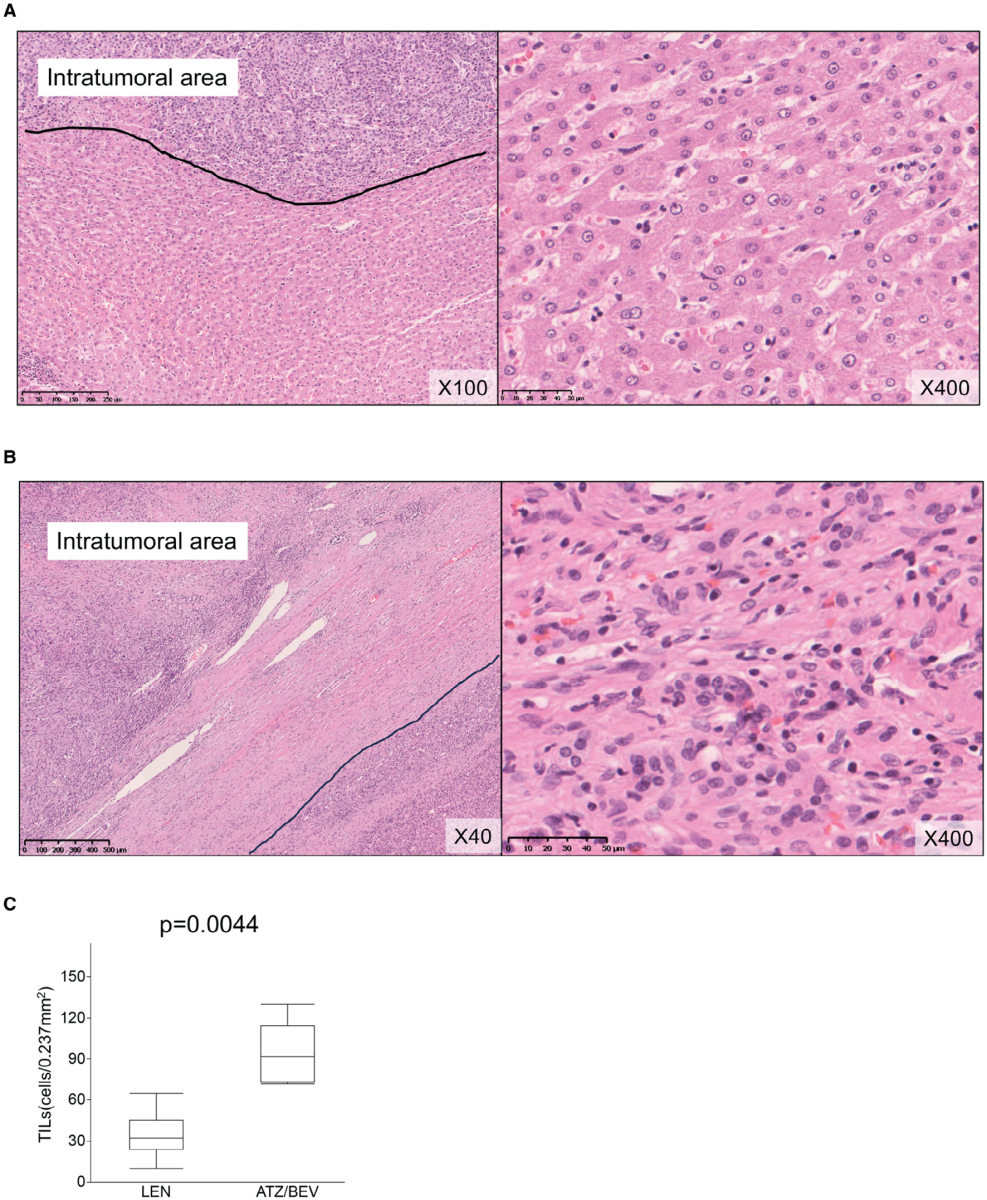
Representative features of TILs in the peritumoral area after LEN (A) and ATZ/BEV (B) therapy. (C) The median (range) TIL counts after LEN and ATZ/BEV therapy were 24 (10–49) and 85 (29–130) cells/0.237 mm^2^, respectively. The number of TILs was significantly higher after ATZ/BEV therapy (*p* = 0.0127). ATZ/BEV, atezolizumab plus bevacizumab; LEN, lenvatinib; TILs, tumor‐infiltrating lymphocytes.

The numbers of TILs/CD3+ TILs/CD8+ TILs in the peritumoral area in resected specimens after each treatment were evaluated. Images of hematoxylin and eosin staining following treatment are presented in Figure 2A,B. The median (range) TIL counts were as follows: LEN, 32 (10–65) cells/0.237 mm^2^; and ATZ/BEV, 92 (72–130) cells/0.237 mm^2^. The number of TILs was significantly higher after ATZ/BEV therapy (*p* = 0.0044, Figure 2C).

**FIGURE 3:**
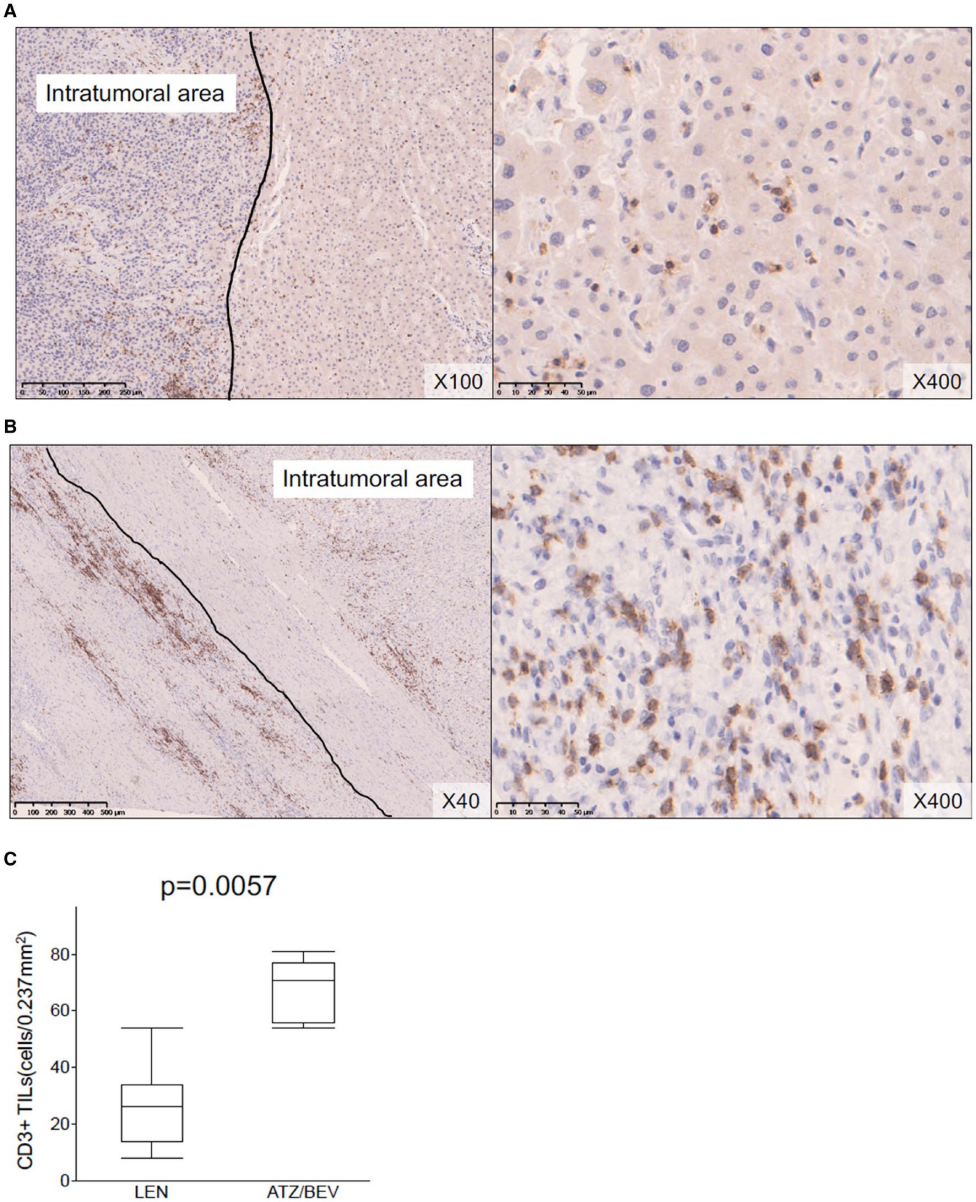
Representative features of CD3+ TILs in the peritumoral area after LEN (A) and ATZ/BEV (B) therapy. (C) The median (range) CD3+ TIL counts after LEN and ATZ/BEV therapy were 23 (8–54) and 73 (33–96) cells/0.237 mm^2^, respectively. The number of CD3+ TILs was significantly higher after ATZ/BEV therapy (*p* = 0.0083). ATZ/BEV, atezolizumab plus bevacizumab; LEN, lenvatinib; TILs, tumor‐infiltrating lymphocytes.

The results of immunohistochemical staining for CD3 are presented in Figure 3A,B. The median (range) CD3+ TIL count was 26 (8–54) cells/0.237 mm^2^ after LEN therapy, versus 71 (54–81) cells/0.237 mm^2^ after ATZ/BEV therapy (*p* = 0.0057, Figure 3C).

**FIGURE 4:**
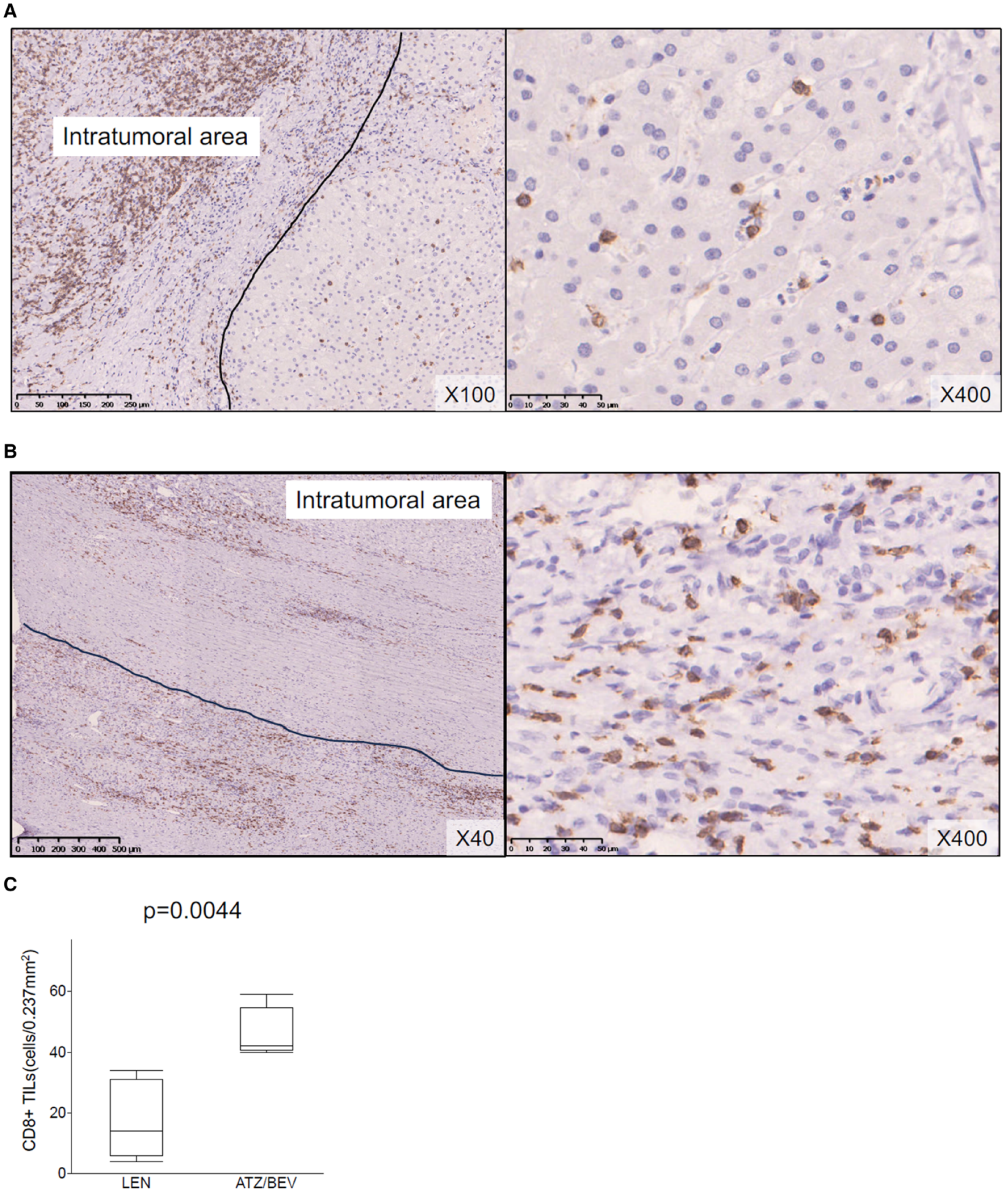
Representative features of CD8+ TILs in the peritumoral area after LEN (A) and ATZ/BEV (B) therapy. (C) The median (range) CD8+ TIL counts after LEN and ATZ/BEV therapy were 13 (4–34) and 41 (17–56) cells/0.237 mm^2^, respectively. The number of CD8+ TILs was significantly higher after ATZ/BEV therapy (*p* = 0.0083). ATZ/BEV, atezolizumab plus bevacizumab; LEN, lenvatinib; TILs, tumor‐infiltrating lymphocytes.

The results of immunohistochemical staining for CD8 are presented in Figure 4A,B. The median (range) CD8+ TIL counts were 14 (4–34) cells/0.237 mm^2^ after LEN therapy and 42 (40–59) cells/0.237 mm^2^ after ATZ/BEV therapy (*p* = 0.0044, Figure 4C). We summarized these results in Table 2.

**TABLE 2:** Evaluation of necrotic areas of the tumor and TIL counts in the peritumoral area in each patient.

Case	Treatment	Tumor size (mm)			Gross classification	Proportion of necrotic area in tumor (%)	TILs count (cells/0.237 mm^2^)	CD3+ TILs count (cells/0.237 mm^2^)	CD8+ TILs count (cells/0.237 mm^2^)
		Before systemic therapy	After systemic therapy	Resected specimen					
1	LEN	20	15	15	Simple nodular type	0	32	26	14
2	LEN + TACE	50	35	30	Not assessable	100	45	34	31
3	LEN	8	9	8	Simple nodular type	0	24	8	6
4	LEN	145	135	140	Simple nodular type	65	10	14	6
5	LEN	58	58	55	Confluent multinodular type	80	33	29	15
6	LEN	70	46	52	Simple nodular type	0	65	54	34
7	LEN + TACE	140	130	135	Simple nodular type	85	24	19	4
8	ATZ/BEV	130	65	58	Simple nodular type	75	130	81	50
9	ATZ/BEV	140	105	102	Not assessable	100	98	73	40
10	ATZ/BEV	70	60	52	Confluent multinodular type	30	74	58	41
11	ATZ/BEV	100	65	70	Simple nodular type	20	72	54	42
12	ATZ/BEV	70	40	40	Not assessable	100	92	71	59

Abbreviations: ATZ/BEV, atezolizumab plus bevacizumab; LEN, lenvatinib; TACE, transcatheter arterial chemoembolization; TILs, tumor‐infiltrating lymphocytes.

### 3.3 Tumor Characteristics of Cases in ATZ/BEV Group

By performing IHC of specimens in ATZ/BEV group, we examined the relationship between the efficacy of ATZ/BEV therapy and tumor characteristics. No pre‐treatment biopsy was performed, so a surgically removed sample was used. One of the five cases showed nuclear translocation of β‐catenin and staining of glutamine synthetase in cytoplasm, but OATP1B3 was not expressed in all cases (Figures S1–S3). In Gd‐EOB‐DTPA‐enhanced MRI before hepatic resection, all five cases showed hypo‐intensity of tumors.

## 4 Discussion

We investigated whether TIL counts in the peritumoral area in patients undergoing hepatic resection differed after LEN and ATZ/BEV therapy and found that TIL, CD3+ TIL, and CD8+ TIL counts were significantly higher after ATZ/BEV therapy.

The TME has important associations with tumor growth, metastatic spread, and treatment responses, and the intra‐tumoral expression profile reflects the interaction between the tumor and immune cells [16, 20, 21]. Meanwhile, a new concept, the peritumor microenvironment (PME), has been developed to explain the mechanisms of the development and progression of HCC, and its profile has also been studied [22]. Yusa et al. reported that immune cells in the peritumoral area of HCC can predict patient prognosis after curative hepatectomy [18]. Recently, there have been significant advances in systemic therapy for HCC, with many researchers focusing on the sensitivity to LEN and ATZ/BEV and the changes in tumor properties induced by these therapies. Studies about the TME and PME in patients undergoing hepatic resection after systemic therapy are limited, and much remains to be elucidated [23]. In this study, we focused on the immune status in the peritumoral area rather than the intra‐tumoral area. The intra‐tumoral evaluation was excluded because TILs could not be properly assessed in cases in which the tumor was partially or mostly necrotic following LEN or ATZ/BEV therapy. Two cases in LEN group underwent TACE, and these cases were also included in the evaluation. The reason for this is that patients who underwent TACE for disease control during LEN therapy may subsequently require surgery. Although both two cases showed extensive necrosis of tumor and could not be accurately evaluated with respect to intra‐tumoral TILs, we considered the importance of reporting information on peritumoral lymphocytic infiltration in these cases in the article. In this study, we demonstrated that TIL/CD3+ TIL/CD8 TIL counts in the peritumoral area were significantly higher after ATZ/BEV therapy than after LEN therapy. In some patients who undergo hepatic resection after systemic therapy, tumor size is relatively large and liver function is often impaired due to systemic therapy, so it is important to determine the surgical technique in consideration of the remnant liver volume. Especially if the tumor is very large, liver resection should be performed as close to the tumor as possible to preserve as much of the remaining liver as possible. In hepatic resection, the liver is basically dissected during the pringle maneuver, but the longer it takes to dissect the liver, the longer the ischemic time of the liver is, which increases the likelihood of elevated serum lactate levels and postoperative liver failure. Therefore, it goes without saying that it should not take too long for the dissection. As Yusa et al. found that a high TIL count in the peritumoral region may be good in terms of controlling tumor progression, but a high TIL count means that inflammation is occurring in the peritumoral region. In solid structure hepatocellular carcinomas, high lymphocyte counts in the peritumoral area have been reported, and if only the peritumoral microenvironment is taken into account, resection after LEN may be connected to a better liver dissection [24].

There was the case which had relatively high peritumoral TILs as case 6 of LEN group in Table 1. Compared to the other cases, case 6 showed no tumor necrosis at all and had a longer treatment period. Although disease control was maintained, it is possible that the process of TILs infiltration into the tumor from the peri0tumoral tumor was observed. On the other hand, there was the case which had small tumor but no necrosis and low peritumoral TILs counts as case 3 of LEN group in Table 1. Although systemic therapy was not highly effective in this case, it was possible that the tumor was of a type that progresses relatively slowly. In the patients included in this study, there were no obvious differences in blood loss or the operative time, as presented in Table 1, attributable to differences in the surgical procedure or patient background. However, one patient each in the LEN and ATZ/BEV group underwent subsequent central bisegmentectomy, and the patient in the LEN group had lower blood loss and lower TIL, CD3+ TIL, and CD8+ TIL counts. Although many studies have reported about biomarkers that predicted response to systemic therapy (LEN, ATZ/BEV) for HCC, no clear criteria have been established for which regimen should be selected and for how long treatment should be performed when surgery is contemplated. When starting systemic therapy with the intention to perform conversion surgery, it is suggested that hepatic resection is less difficult after LEN therapy because of the lower TIL counts and less severe inflammation in the peritumoral area. Depending on the patient's condition, if the tumor is large and there is a need to proceed with hepatic resection close to the tumor, it is suggested that hepatic resection is less difficult after LEN therapy because of the lower TIL counts and less severe inflammation in the peritumoral area.

Although intra‐tumor vascular characteristics, such as vessels encapsulating tumor clusters, influence sensitivity to systemic therapy, one limitation this study is that it only included surgically removed samples and did not assess tumor characteristics or peritumor TIL prior to systemic therapy. Further study with higher numbers of patients is needed.

In conclusion, the numbers of TILs, CD3+ TILs, and CD8+ TILs in the peritumoral area were significantly higher after ATZ/BEV than after LEN therapy.

## Author Contributions

**Katsuya Toshida:** conceptualization (supporting), data curation (supporting), formal analysis (supporting), funding acquisition (supporting), investigation (supporting), methodology (supporting), project administration (supporting), resources (supporting), software (supporting), supervision (supporting), validation (supporting), visualization (supporting), writing – original draft (lead), writing – review and editing (supporting). **Shinji Itoh:** conceptualization (lead), data curation (lead), formal analysis (lead), funding acquisition (lead), investigation (lead), methodology (lead), project administration (lead), resources (lead), software (lead), supervision (lead), validation (lead), visualization (lead), writing – original draft (supporting), writing – review and editing (supporting). **Yasushi Tanaka:** investigation (supporting). **Takeo Toshima:** investigation (supporting). **Shohei Yoshiya:** investigation (supporting). **Takuma Izumi:** investigation (supporting). **Norifumi Iseda:** investigation (supporting). **Yuriko Tsutsui:** investigation (supporting). **Yuki Nakayama:** investigation (supporting). **Takuma Ishikawa:** investigation (equal). **Mizuki Ninomiya:** investigation (supporting). **Takeshi Iwasaki:** investigation (supporting). **Yoshinao Oda:** writing – review and editing (lead). **Tomoharu Yoshizumi:** writing – review and editing (lead).

## Ethics Statement

This retrospective study was approved by the ethics committee of Kyushu University Hospital (number: 23029–00). We obtained informed consent from all patients.

## Conflicts of Interest

Tomoharu Yoshizumi has received lecture fees from Eisai Co. Ltd. and Chugai Pharmaceutical Co. Ltd. All other authors have no conflicts of interest to declare.

## Supporting information


**Figure S1.** Representative features of β‐catenin. Left: β‐catenin expression in nucleus. Right: β‐catenin expression in cell membrane.
**Figure S2.** Representative features of glutamine synthetase. Left: No expression of glutamine synthetase. Right: Expression of glutamine synthetase in cytoplasm.
**Figure S3.** Representative features of OATP1B3. Left: No expression of glutamine synthetase. Right: Expression of OATP1B3 in cell membrane of normal liver.

## Data Availability

The data are not publicly available due to containing information that could compromise the privacy of research participants.
