# Supplementary material for: Differences in Tumor‐Infiltrating Lymphocyte Counts in the Peritumoral Area in Patients Undergoing Hepatic Resection After Lenvatinib and Atezolizumab Plus Bevacizumab Therapy for Hepatocellular Carcinoma
Source: Cancer Med. 2025 Apr 18;14(8):e70445. doi: 10.1002/cam4.70445 (PMC12007420; doi:10.1002/cam4.70445)
Supplement: Supplementary file 1 — Figure S1. Representative features of β‐catenin. Left: β‐catenin expression in nucleus. Right: β‐catenin expression in cell membrane. Figure S2. Representative features of glutamine synthetase. Left: No expression of glutamine synthetase. Right: Expression of glutamine synthetase in cytoplasm. Figure S3. Representative features of OATP1B3. Left: No expression of glutamine synthetase. Right: Expression of OATP1B3 in cell membrane of normal liver. [file CAM4-14-e70445-s001.zip › Figure(supple)_0806KT.pptx]

## Slide 1
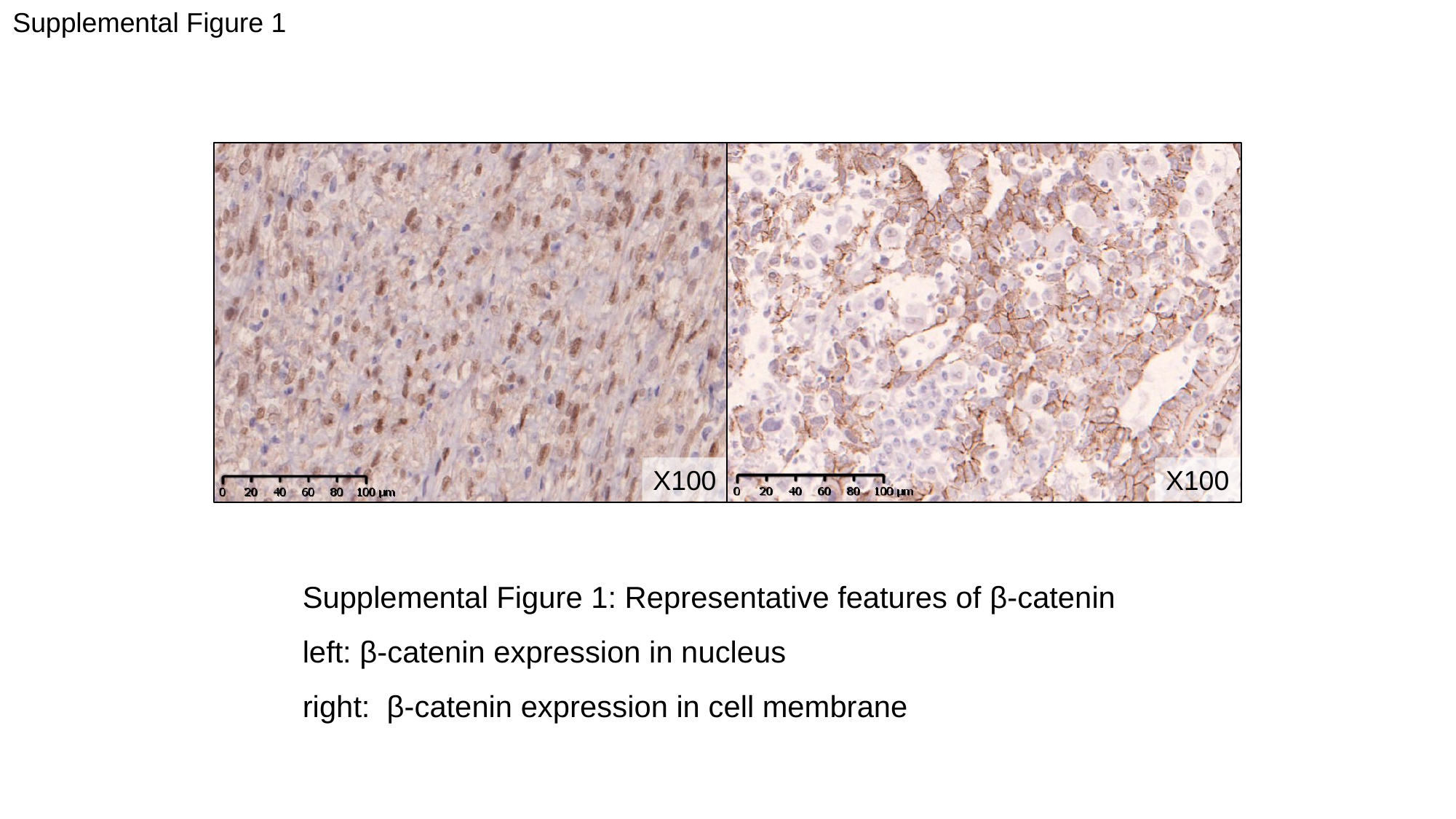

Supplemental Figure 1
X100
X100
Supplemental Figure 1: Representative features of β-catenin
left: β-catenin expression in nucleus
right: β-catenin expression in cell membrane

## Slide 2
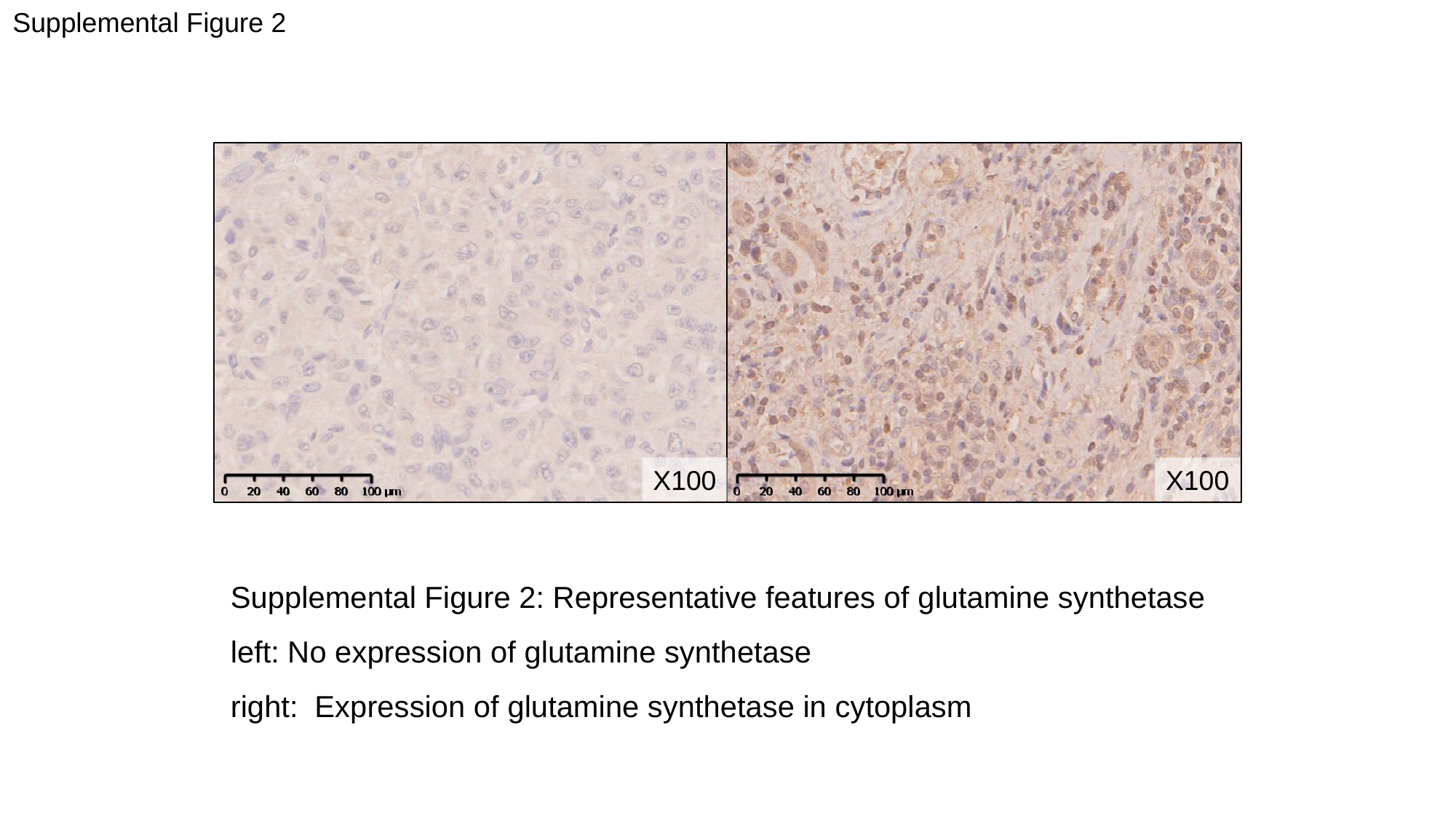

Supplemental Figure 2
X100
X100
Supplemental Figure 2: Representative features of glutamine synthetase
left: No expression of glutamine synthetase
right: Expression of glutamine synthetase in cytoplasm

## Slide 3
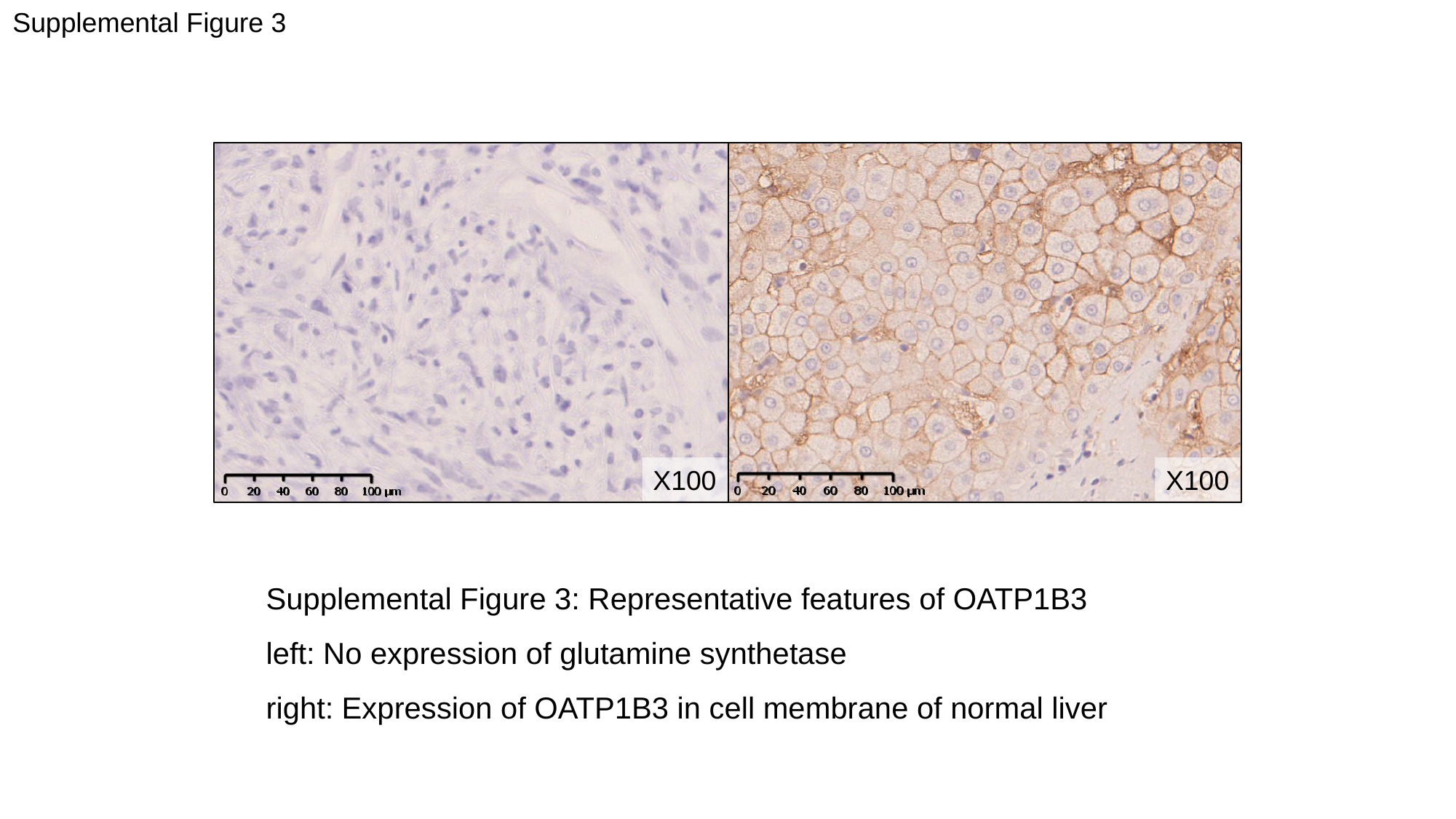

Supplemental Figure 3
X100
X100
Supplemental Figure 3: Representative features of OATP1B3
left: No expression of glutamine synthetase
right: Expression of OATP1B3 in cell membrane of normal liver
