# Supplementary material for: Differences in Tumor‐Infiltrating Lymphocyte Counts in the Peritumoral Area in Patients Undergoing Hepatic Resection After Lenvatinib and Atezolizumab Plus Bevacizumab Therapy for Hepatocellular Carcinoma
Source: Cancer Med. 2025 Apr 18;14(8):e70445. doi: 10.1002/cam4.70445 (PMC12007420; doi:10.1002/cam4.70445)

Supplemental Table 1. Prioperative biomarkers of patients who underwent hepatic resection

| Case | Treatment | Best response:  RECIST  modified-RECIST | NLR | LMR | NMR | LCR |
| --- | --- | --- | --- | --- | --- | --- |
| 1 | LEN | PR  PR | 2.41 | 3.08 | 7.48 | 6544 |
| 2 | LEN+TACE | PR  PR | 1.04 | 6.55 | 6.79 | 37642 |
| 3 | LEN | PR  PR | 2.25 | 2.47 | 5.57 | 53550 |
| 4 | LEN | PR  PR | 2.97 | 3.45 | 10.3 | 509 |
| 5 | LEN | SD  PR | 3.20 | 4.17 | 13.4 | 459 |
| 6 | LEN | PR  PR | 1.64 | 10.3 | 16.9 | 25916 |
| 7 | LEN+TACE | SD  PR | 3.26 | 3.16 | 10.3 | 397 |
| 8 | ATZ/BEV | PR  PR | 2.33 | 3.89 | 9.09 | 8063. |
| 9 | ATZ/BEV | PR  CR | 3.69 | 1.95 | 7.23 | 10422 |
| 10 | ATZ/BEV | PR  PR | 4.92 | 2.11 | 10.4 | 5585 |
| 11 | ATZ/BEV | PR  PR | 3.04 | 2.14 | 6.51 | 2415 |
| 12 | ATZ/BEV | PR  CR | 0.75 | 9.87 | 7.43 | 8784 |

LEN, lenvatinib; ATZ/BEV, atezolizumab plus bevacizumab; TACE, transcatheter arterial chemoembolization; LMR, lymphocyte-monocyte ratio; NLR, neutrophil-lymphocyte ratio, NMR, neutrophil-monocyte ratio; RECIST, Response Evaluation Criteria in Solid Tumors; PR, partial response; CR, complete response


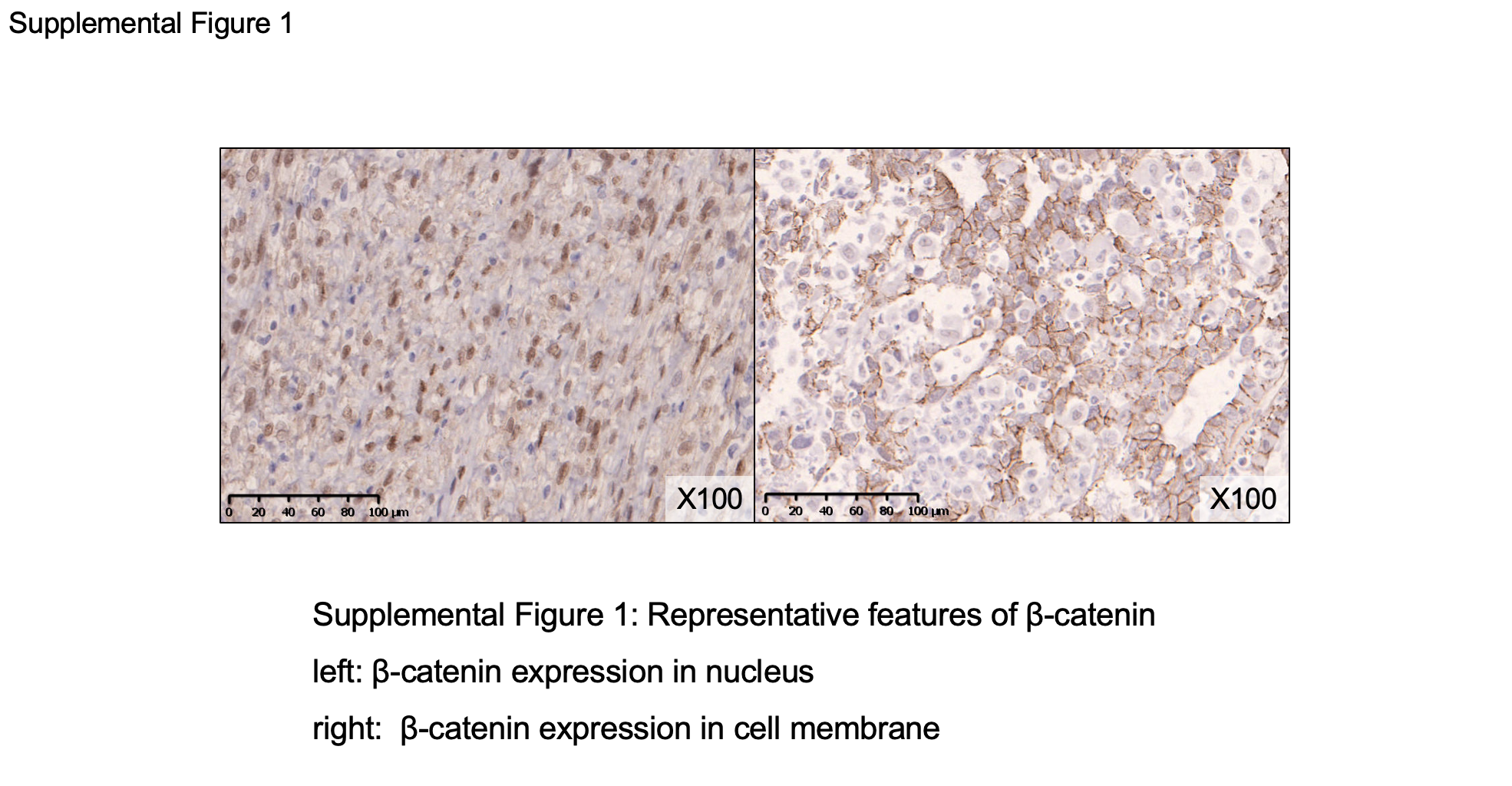


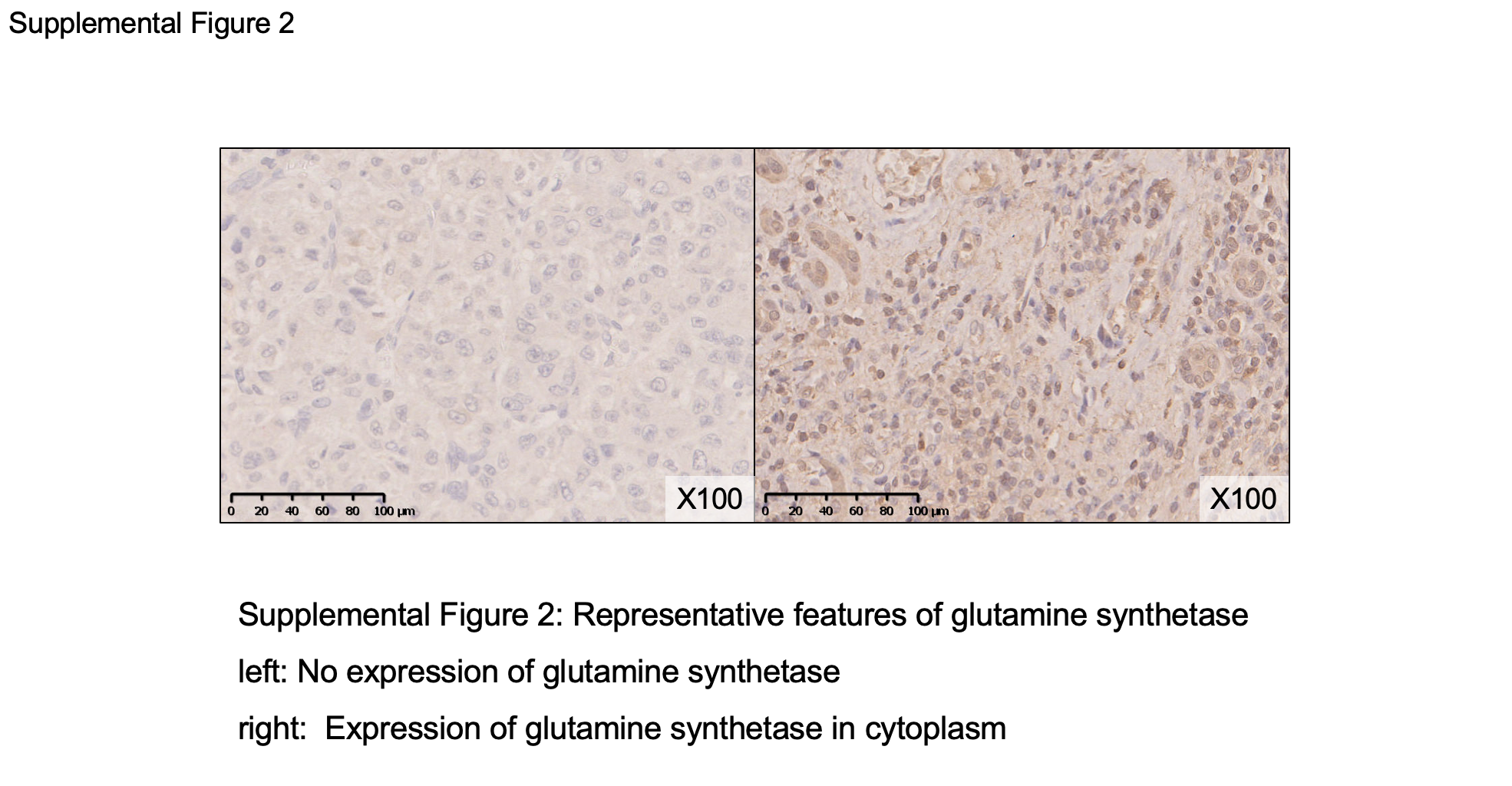


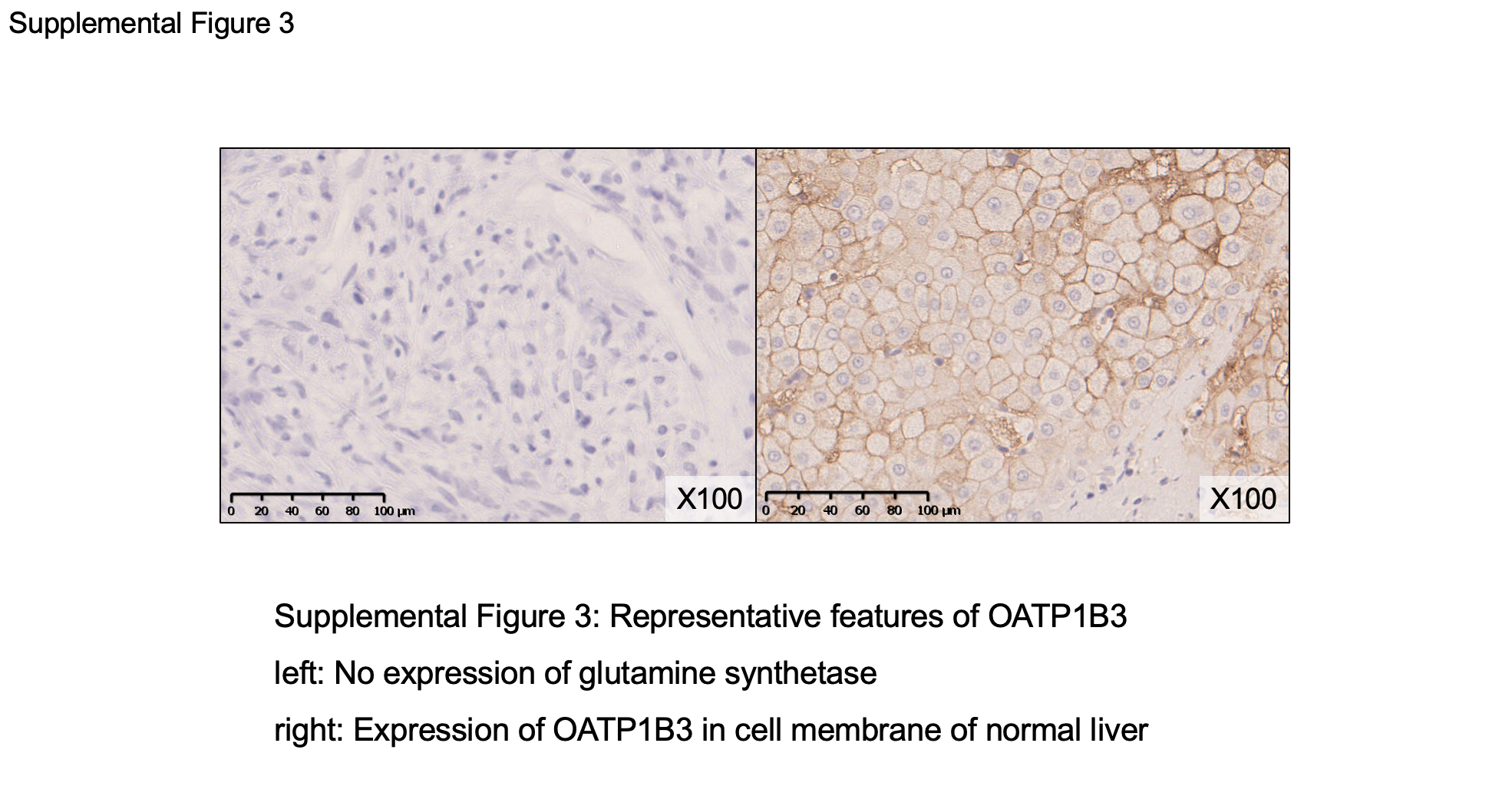

Supplement: Supplementary file 1 — Figure S1. Representative features of β‐catenin. Left: β‐catenin expression in nucleus. Right: β‐catenin expression in cell membrane. Figure S2. Representative features of glutamine synthetase. Left: No expression of glutamine synthetase. Right: Expression of glutamine synthetase in cytoplasm. Figure S3. Representative features of OATP1B3. Left: No expression of glutamine synthetase. Right: Expression of OATP1B3 in cell membrane of normal liver. [file CAM4-14-e70445-s001.zip › Supplementary material_20240922.docx]
